# Enormous Exostosis of the Sternum Converted into Adipocere

**Published:** 1829-01-01

**Authors:** 


### HOSPITAL OF SURGERY.

I. EXOSTOSIS OF THE STERNUM CONVERTED INTO ADIPOCERE.

In the month of October, 1827, the following short notice of a "*case of enor-*

*mous exostosis of the sternum,*" was reported from the HOSPITAL of SURGERY, in Panton Sqare.

"———, about fifty years of age, was sent to the Hospital by Dr. Scott, of Barnes. Arising by a very broad attachment, from nearly the whole of the sternum, except the superior part of its upper bone, adhering to the cartilages of the ribs, and extending so far outwards as to elevate the papillæ, is a very large osseous tumour, measuring eighteen inches in circumference; its surface, which is smooth, and not marked by any of those protuberances which were a distinguishing feature in the case of osteosarcoma, *is evidently composed throughout of the same solid materials,* and does not afford to the finger that feeling of elasticity which was experienced in the other, at those places where the fleshy intermixture existed. The integuments covering the swelling are much stretched, and have become, at the most protuberant point, inflamed.—The disease is attended with little pain, and is principally inconvenient from its size and weight. His general health is in a very indifferent state; he is dyspeptic, subject to rheumatic attacks, and occasionally suffers from dyspnœa.

"'The tumour commenced nine years ago, without any apparent cause, with general enlargement of the sternum at that surface where it is now attached, which has gradually increased, till it has assumed its present immense size.'"

In September, 1828, this patient died, and Mr. Parker, of Woburn, had an opportunity of examining the body. The following statement will shew, that *physic* is not the only conjectural art in this world, but that surgery sometimes comes in for a share.

"The tumour, on examination, *possessed no character of exostosis,* or *osteosarcoma;* throughout, its texture was soft, though solid, and appeared to have been well supplied with vessels. To give a familiar idea of its appearance and consistency, it very much resembled adipocere, except in colour, which was, for the most part, of a dingy red. On dissecting back the integument on either side, it was observed, that the left pectoral muscle was remarkably pallid and attenuated; the muscle of the opposite side presented nothing remarkable. The tumour was covered with a thin layer of adeps, without the appearance of any distinct capsule or investment. Supposing it to have originated from the sternum, I attempted to dissect it off entire, but finding it more deeply imbedded than the situation of the bone would explain, I opened the thorax in the usual way, expecting that the extent and connexions of the disease would be at once developed. This was by no means the case, and to satisfy myself as fully as possible, I extended the opening to the parietes of the abdomen, and thus found the apex of the tumour projecting with the diaphragm before it, within an inch of the umbilicus. Without disturbing any of the viscera, I endeavoured to trace it through its whole extent; this I was in some measure enabled to do, though not completely to my satisfaction. On passing my hand between the tumour and the arch of the ribs, considerable resistance was offered from adhesions, apparently of long standing. Having accomplished its detachment on both sides, as well as the upper part, which was also adherent in a slighter degree, I continued the examination to the diaphragm; here the tumour was in close contact with the tendon of that muscle, indeed inseparably so, and on detaching it, which I could do in no other way than by cutting through the diaphragm, I found the heart healthy, but small and compressed, without its pericardium, immediately behind the tumour. The lungs were much smaller than natural, and flattened, evidently from want of space. From the examination, it appears to me, that the growth of the disease must have begun in the pericardium, and this opinion is strengthened, from the circumstance of the tumour having a distinct investment, answering to the pericardium on its under and posterior surface. The sternum was completely absorbed, not even a vestige of it remaining, and the cartilages of the ribs were unusually soft in a man approaching to 60."

Well might Mr. Parker say "unfortunately for surgery, this case is strikingly illustrative of the fallibility of human judgment." Yes! it ought to lower the pride, and increase the charity of medical men towards their brethren! Unfortunately, none are more forward to accuse others of mistakes and errors than those who, themselves, are every day falling into them ! The candid confession of the errors in *diagnosis*, which *all* medical men are constantly making, but which so few acknowledge, would do immense service, both in a moral and physical point of view !

